# Vertical Paper Analytical Devices Fabricated Using the Principles of Quilling and Kirigami

**DOI:** 10.1038/s41598-017-07267-9

**Published:** 2017-08-03

**Authors:** Bingbing Gao, Junjie Chi, Hong Liu, Zhongze Gu

**Affiliations:** 1 State Key Laboratory of Bioelectronics, School of Biological Science and Medical Engineering, Southeast University, Nanjing, 210096 China; 2 Laboratory of Environment and Biosafety, Research Institute of Southeast University in Suzhou, Suzhou, 215123 China

## Abstract

Here we report the vertical paper analytical devices (*v*PADs) fabricated using the principles of quilling and kirigami. What differentiates the *v*PADs from conventional paper microfluidic devices is that the paper substrate used to fabricate the device is placed vertically to the device plane. The fabrication of *v*PADs with high precision is instrument-free, requiring no photolithography, printing or heating. Two- and three-dimensional *v*PADs are fabricated for multiplex colorimetric assays of four biochemical indicators and automated enzyme-linked immunosorbent assay of human myoglobin, respectively.

## Introduction

Paper-based analytical devices such as dipsticks and lateral-flow test strips have long been used for point-of-care diagnostics because they are fast, inexpensive and easy-to-use. In 2007, Whitesides *et al*. reported microfluidic paper-based analytical devices (μPADs) based on two-dimensional (2D) and three-dimensional (3D) paper capillary channels patterned using photolithography^1^. After that, many μPADs with interesting designs and functions have been presented, such as origami-inspired μPADs (oPADs)^2, 3^ and slipPADs^4^. Paper-based analytical devices have found a wide range of applications in multiplex, power-free, on-site chemical analysis, particularly in underdeveloped areas. Various methods for patterning paper have been reported, including photolithography^1^, plotting^5^, cutting^6^, inkjet printing^7^ and wax printing^8^. These methods selectively deliver energy, materials, or both onto a sheet of paper plane to create capillary channels. Due to the inhomogeneity of cellulose paper, the lateral resolution of the channel is larger than 500 μm even using sophisticated photolithography. Instrumental-free fabrication of 2D and 3D devices with acceptable channel precision is still challenging.

Quilling, which dates back to at least the 13th Century, is a form of art involving the use of paper strips that are rolled and glued together to create decorative designs^9, 10^. As another type of paper art, kirigami is a variation of origami that includes cutting of the paper rather than just paper folding^11^. The μPADs we report here are inspired by these two types of paper arts. They are named vertical paper analytical devices (vPADs) because the paper strips used to fabricate the device are placed vertically to the device plane. The 2D vPADs are fabricated by rolling paper strips around a slotted stick. Double-sided adhesive tape is attached to both sides of the paper strips for assembly of the device and also used as water-proof channel barriers. Aqueous solution can then wick through the channel by capillary action. 3D vPADs are fabricated using the principles of both kirigami and quilling. Paper strips are first cut and then assembled into 3D devices. For the proof-of-concept, multiplex colorimetric assays and automated enzyme-linked immunosorbent assay (ELISA) are carried out on the vPADs.

The vPADs have several interesting characteristics that are potentially useful. First, unlike most paper microfluidic device, the fabrication of vPADs is instrument-free. No photolithography, printing or heating is required^6, 7, 12–16^. This is useful for point-of-care diagnostics in developing countries which have infrastructure shortfalls such as unreliable electric power and poor maintenance for sophisticated instruments^17^. Second, the channel width of vPADs is determined by the thickness of the paper. So the fabrication of channels from 0.07 mm to 1 mm with high precision is straightforward using commercially available paper, tape and hands^18^. Third, channels are rolled into a small area on the chip. Using paper strips having a thickness of 80 μm, one can estimate that the maximum length of channel per unit area is about 20 cm/cm^2^ for a 2D vPAD. This can be useful for high-throughput and/or multiplex assays which require high channel density on a single chip^19^. Forth, because the paper strips are vertical to the chip plane, the use of paper strips that are patterned will result in 3D devices with 3D channel network for carrying out complicated bioassays^1, 2^. Fifth, the colour observed vertically to the paper plane is deeper due to increased optical path, which leads to a higher sensitivity for colorimetric detection. Finally, because only the edge of the channel is exposed to the air, the problems for conventional devices such as water evaporation and contamination can be easily solved^3^.

## Results and Discussion

**Figure 1:**
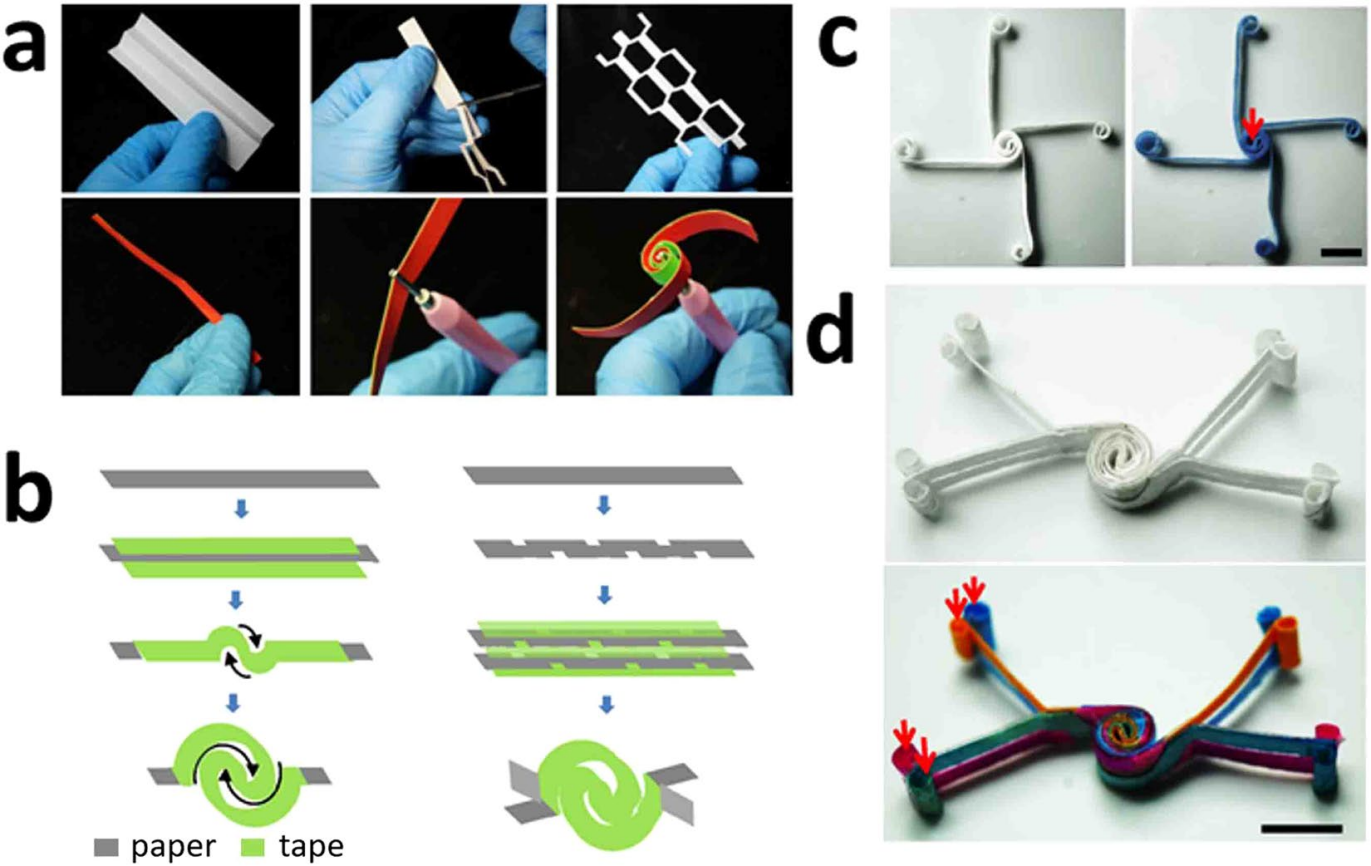
(**a**) Photographs showing the quilling and kirigami processes. (**b**) Schematic illustration showing the procedure for fabricating the 2D and 3D vPADs using the principles of quilling and kirigami. (**c**) Photographs of the 2D vPAD before and after the introduction of 0.10 M PBS solution containing 0.010 M coomassie brilliant blue. The inlet for introduction of the solution is indicated by the red arrow. Scale bar: 10 mm. (**d**) Photographs of the 3D vPAD before and after the introduction of 0.10 M PBS solutions containing 0.010 M coomassie brilliant blue, 0.010 M rhodamine B, 0.010 M methyl orange and 0.010 M bromocresol green, respectively. The inlets for introduction of the solutions are indicated by the red arrows. Scale bar: 10 mm.

Paper-based analytical devices, including dipsticks, lateral-flow test strips and μPADs have been widely used in biochemical analysis. In this work, we introduce another type of μPADs, which are intrinsically different from conventional μPADs. The basic processes of kirigami and quilling processes are shown in Fig. 1a. For kirigami, a sheet of paper was folded, cut, and finally unfolded to obtain a sheet of patterned paper, one can easily get several duplicated patterns by single cutting. The cutting with scissors could also be replaced by automated cutting machine such as a laser cutter to get more precise paper patterns. For quilling, double-sided adhesive tapes were attached onto both sides of the paper strip, and the paper strip was rolled to assemble the device. Using the principles of kirigami and quilling, we fabricated basic 2D and 3D vPADs as shown in Fig. 1b. For the 2D vPAD (Fig. 1c), the double-sided tapes were attached on both sides of the paper strip not only for assembly of the device, but also used as water-proof channel barriers to avoid the mixing of solutions in different channels. For the 3D vPAD (Fig. 1d), the paper strip was first patterned using scissors and then assembled into the 3D device.

The 2D and 3D vPADs fabricated were used to demonstrate the ability of the devices to direct the flow of fluids in two and three dimensions. Specifically, 0.10 M PBS solution (pH 7.4) containing 0.010 M coomassie brilliant blue was dropcast onto the inlet of the 2D device (Fig. 1c), the blue-colored solution wicked evenly through their designated channels without mixing with other channels. For 3D vPADs, 0.10 M PBS solutions (pH 7.4) containing 0.010 M of the following dyes were dropcast onto the inlets of the device (indicated by the red arrows), respectively: coomassie brilliant blue (blue), rhodamine B (red), methyl orange (orange) and bromocresol green (green). The dye solutions flowed through the entire separated channels evenly without mixing or leaking.

**Figure 2:**
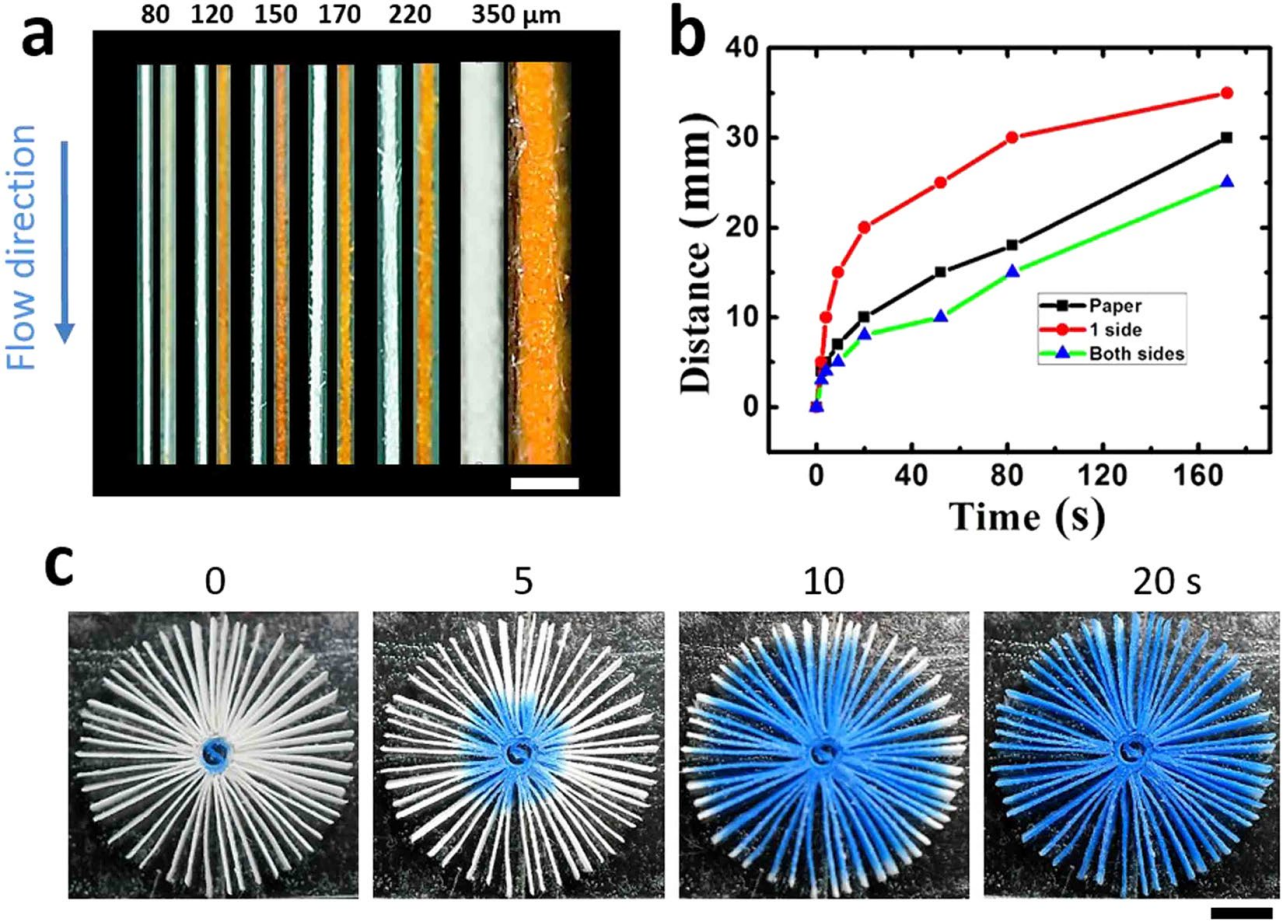
(**a**) Photographs of paper channels fabricated using paper stirps with different widths before and after introduction of an aqueous solution containing 10 mM methyl orange. The width of the paper strips are indicated. (**b**) Distance travelled by the solution as a function of time for different paper channels. (**c**) Photographs of a vPAD device after introduction of an aqueous solution containing 10 mM coomassie brilliant blue.

In principle, the resolution of vPAD channels was determined by the thickness of paper strips. As the demonstration, six types of paper strips with different thicknesses ranging from 80–350 μm were obtained, and used to fabricate the vPAD. As shown in Fig. 2a, a dye solution was used to test the liquid flow through each channel. We measured the flow length of an aqueous 0.010 M coomassie brilliant blue solution in three types of paper channels as a function of time: (1) only paper; (2) paper having one side attached with tape; (3) paper channel having both sides attached with tapes. The results showed that tapes slowed down the flow of solution through the capillary channel, which is reasonable because the tape is less hydrophilic than paper. The channel width of the vPAD is determined by the thickness of paper and tape. Therefore, by using really thin paper strips, large number of channels can be integrated in a device with a small area. As shown in Fig. 2c, we can fabricate a vPAD with 50 parallel channels which were all connected to one sample inlet. This device could be potentially useful for highthroughput and multiplex assays.

**Figure 3:**
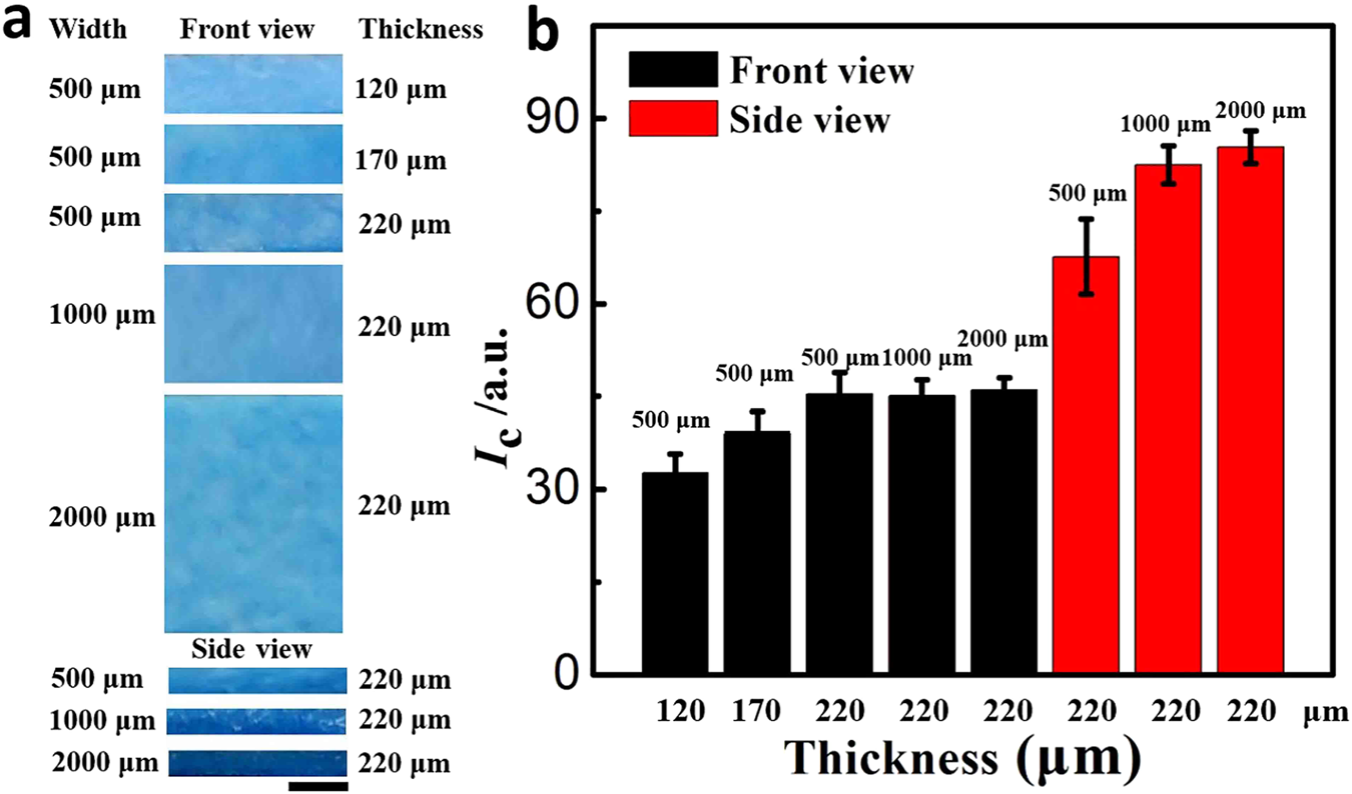
(**a**) Photographs showing the side and front views of three types of paper channels after introduction of 0.010 M coomassie brilliant blue solution. The widths and thickness of the paper strips used to fabricate the channel are indicated. Scale bar: 0.5 mm (**b**) Color intensity (*I*c) of the channels measured from these photographs.

When dye solution infiltrated through paper channels, uniformly dispersed dye molecules gradually concentrated at the edge of the paper as water evaporates, known as the coffee ring effect. More dye molecules aggregated alone the edge compared with intermediate zone. Therefore the colour intensity of dyes observed from the side view should be much higher than that from the front view, which could be used to increase the sensitivity for colorimetric detection. We fabricated paper channels with different widths ranging from 500–2000 μm, as shown in Fig. 3a. After introduction of solution containing 0.010 M coomassie brilliant blue, the optical photographs of front and side views of the paper channels were collected. The colour intensity of each channel was obtained from a histogram of the channel after imported into Adobe Photoshop CS5. As shown in Fig. 3b, the colour intensity of the channel measured from the side view was obviously higher than that measured from the front view. The colour intensity of the front view increased with the thickness of the paper strips, which means longer optical path resulted in higher colour intensity. The results indicated that vPAD channels can be used to increase the detection signal of colorimetric assays.

**Figure 4:**
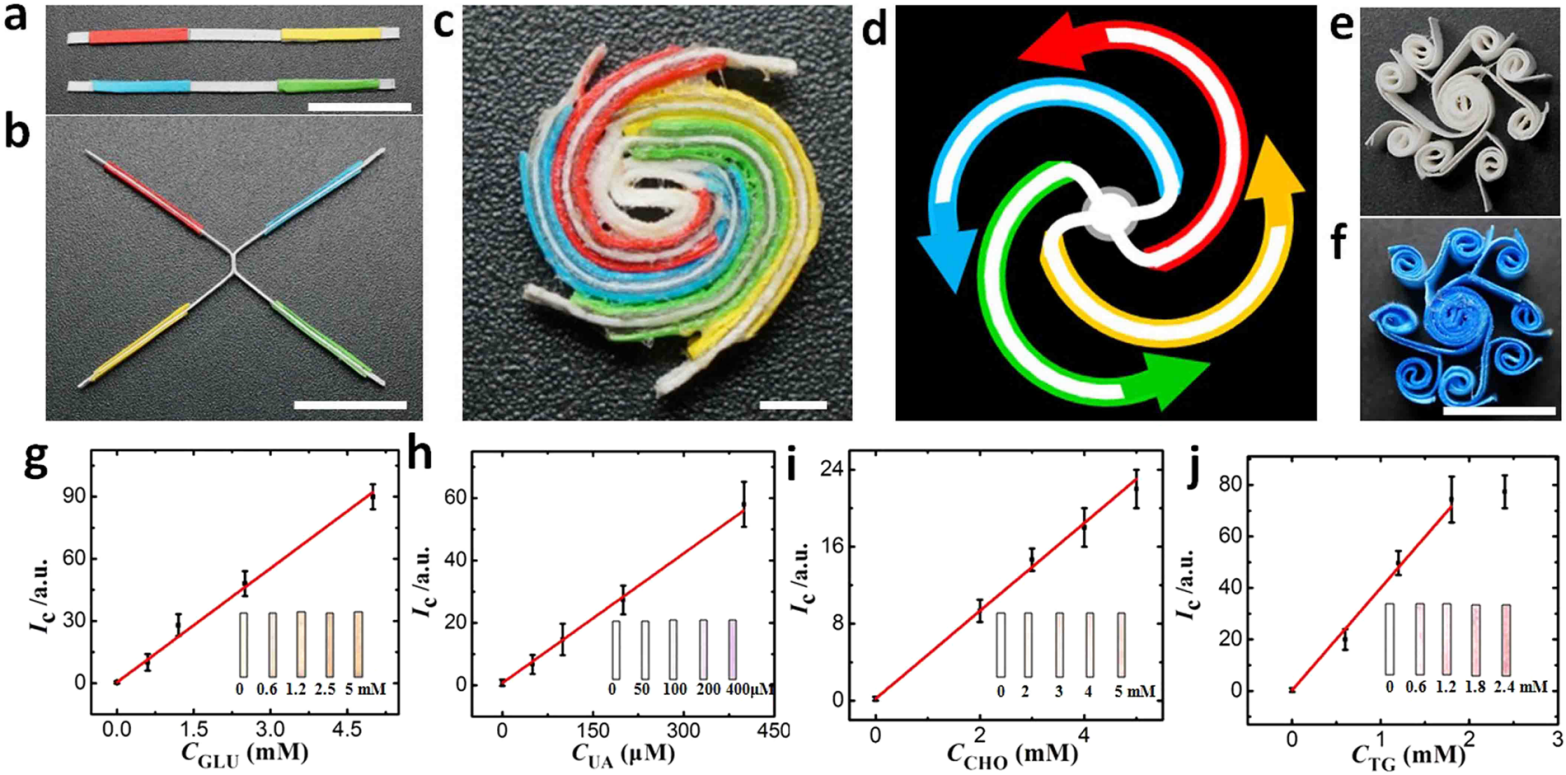
(**a**,**b**) Photographs of the paper strips attached with tapes for the device fabrication. (**c**) The resulted 2D vPAD for colorimetric detection of four analytes (GLU: glucose, UA: uric acid, CHO: cholesterol, TG: triglycerides), scale bar: 2 mm. The colors of the paper strips are from the double-sided adhesive tapes used to assemble the device. (**d**) The resulted fluidic channels and flow direction (indicated by arrows) of aqueous sample in the device. (**e**) The vPAD for carrying out eight parallel assays. (**f**) The vPAD after introduction of 0.010 M coomassie brilliant blue solution. The assays results measured from the 2D vPAD for colorimetric detection of four analytes: (**g**) GLU: glucose; (**h**) UA: uric acid; (**i**) CHO: cholesterol; and (**j**) TG: triglycerides. The arrow bars indicate the standard deviation of three replicated measurements.

To demonstrate the applicability of vPADs to multiplex biochemical analysis, we first fabricated a 2D vPADs for multiplex colorimetric detection of four analytes (glucose, uric acid, cholesterol and triglyceride) which are important biochemical indicators of human health. The multiplex assays were carried out on the 2D vPADs shown in Fig. 4. Two paper strips preloaded with assay reagents were used to fabricate the vPAD. Double-sided adhesive tapes of four different colours were attached to the paper strips for assembly of the device, and the colour of the tape indicated the target being analysed in each channel (red: glucose, blue: uric acid, yellow: cholesterol, green: triglyceride). It is worth noting that, multiplex assays for eight analytes can also be simply carried out on the vPADs by fabricating eight channels using the proposed method, as shown in Fig. 4e,f.

The vPAD can be used for multiplex detection of analytes. Four important biochemical indicators (i.e glucose, uric acid, cholesterol and triglyceride) were used as the model analytes because they are widely used for diagnostics. For example, blood glucose was used for diabetes diagnostics, uric acid is a metabolic product of purine nucleotides which is associated with gout, diabetes and kidney stones, cholesterol is usually used for screening of atherosclerotic risk and lipid metabolic disorders, and triglycerides are the main constituent of human body fat and therefore utilized in the diagnosis and treatment of patients having diabetes mellitus, nephritis, liver obstruction, lipid metabolism disorders and numerous other endocrine diseases. For these biochemical species, the normal blood levels are 3.9–5.5 mM for glucose (tested while fasting)^20^, 0.20–0.430 mM (men) and 0.14–0.36 mM (women) for uric acid^21, 22^, 2.9–6.0 mM for total cholesterol^23, 24^, and 0.56–1.7 mM for triglycerides^25, 26^.

As shown in Fig. 4, the colour was developed in each detection reservoir after reaction of 20 min. The intensity of the color change was correlated to the target concentration. To better quantify these results, the optical photograph of each reservoir was obtained using an office scanner and imported into Adobe Photoshop CS5. The colour intensity was obtained from a histogram of the photograph. As shown in Fig. 4g, the intensity of the brown colour was linearly correlated to the glucose concentration from 0.60 to 5.0 mM (*I*c = 0.43 + 18 × C_GLU_). For uric acid, the intensity of the purple colour was linearly correlated to the concentration of uric acid from 50 to 400 µM (*I*c = 0.68 + 0.14 × *C*_UA_) as showed in Fig. 4h. Figure 4i showed the intensity of the brown color was linearly correlated to cholesterol concentration from 2.0 to 5.0 mM (*I*c = 0.21 + 4.6 × *C*_CHO_). For triglycerides, the intensity of the purple colour was linearly correlated to the triglyceride concentration from 0.60 to 2.4 mM (*I*c = 0.25 + 40 × *C*_TG_), as showed in Fig. 4j. The limit of detections, calculated as 3 times the standard deviation of the sample containing no analyte divided by the slope of the calibration curve, were 0.42 mM for glucose, 35 µM for uric acid, 0.55 mM for cholesterol and 0.32 mM for triglyceride, respectively.

**Figure 5:**
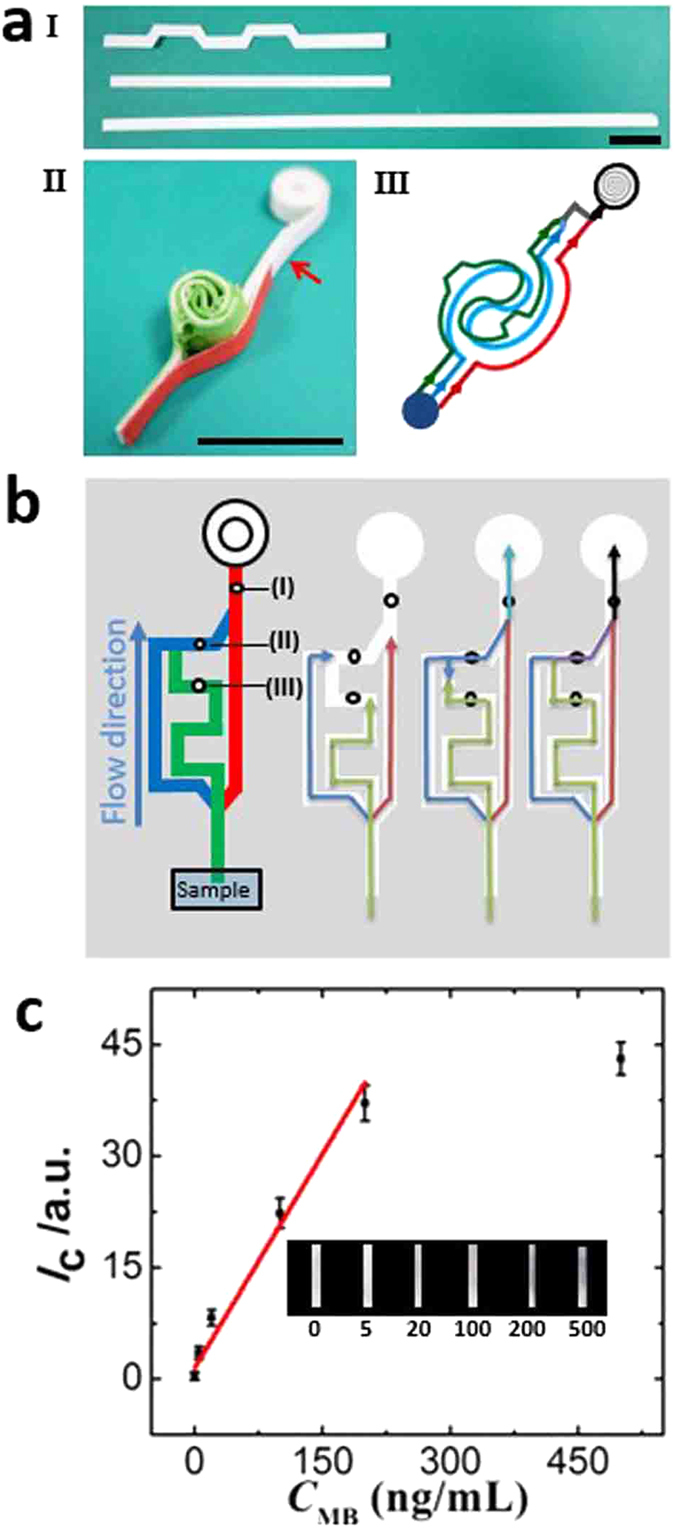
(**a**) Photographs of the paper strips (I) for the device fabrication and the resulted 3D vPAD (II) for myoglobin ELISA. The colors of the paper strips are from the double-sided adhesive tapes used to assemble the device. III: the resulted fluidic channels and flow direction (indicated by arrows) of aqueous sample in the device. Scale bars: 10 mm. (**b**) Schematic illustration showing the flow direction of aqueous sample in the 3D vPAD during the ELISA of myoglobin. Assays reagents (i.e. I: anti-human myoglobin antibody, II: ALP-labelled anti human myoglobin antibody, III: BCIP/NBT substrate) were preloaded on the test strips. (**c**) Color intensity (*I*c) of the detection reservoir measured after completion of the assay as a function of myoglobin concentration (CMB) in the sample. The error bars represent standard deviation of 3 replicate measurements. Inset: photographs of the detection reservoirs. The concentration of myoglobin in the sample is indicated below each photograph.

To demonstrate the applicability of the vPAD to more complex assays, a 3D vPAD was fabricated for automated ELISA^27^ of human myoglobin (Fig. 5a). Myoglobin is a protein found in the muscle tissue of human. It is only found in the bloodstream after muscle injury, so the detection of blood myoglobin is used for diagnostics of injury. The 3D vPAD for ELISA was fabricated using one NC strip and two paper strips, which were blocked with the blocking solution. 1.0 µL of 0.50 mg/mL anti-human myoglobin antibody solution was transferred to location I of the NC strip (Fig. 5b) and then the NC strip was blocked with the blocking solution. ALP-labelled anti-human myoglobin antibody was dropcast into location II of one paper strip. BCIP/NBT substrate was preloaded onto the other paper strip at location III. The detection reservoir was indicated by red arrow in Fig. 5a. After introduction of aqueous sample from the device inlet, the aqueous sample flowed through the 3D channel and initiate the assay process, which included the capture of myoglobin by the antibody immobilized in location I, the binding of ALP-labelled antibody with the captured myoglobin, the washing of the excess reagents and the ALP catalyzed reaction involving BCIP/NBT for color generation. The four steps of the assay accomplished sequentially in the channel. Finally, the color developed in the detection reservoir was measured and correlated to the myoglobin concentration in the sample, as shown in Fig. 5c. For quantitative detection, the color intensity was plotted as a function of the myoglobin concentration. As shown in Fig. 5c, the intensity of the color was linearly correlated to the concentration from 0.050 to 2.0 µg/mL (*I*c = 1.5 + 0.19 × *C*_MB_). The limit of detection, calculated as 3 times the standard deviation of the sample containing no analyte divided by the slope of the calibration curve, was 32 ng/mL.

In conclusion, we have reported the vPADs for chemical analysis under resource-limited conditions. The fabrication of vPADs was based on the principles of quilling and kirigami, and thus the paper substrate used to fabricate the device was vertical to the device plane. The fabrication of vPADs with high precision was instrument-free, requiring no photolithography, printing or heating. To demonstrate the applicability of vPADs to chemical detection under resource-limited settings, 2D and 3D vPADs were fabricated and used for multiplex colorimetric assays of four biochemical indicators and for automated ELISA of human myoglobin, respectively. However the vPADs still have some limitations. For example, the time required for fabrication is still long compared with conventional paper devices due to manual operation. Reproducible production on a large scale of the device will still rely on instruments. The height of the channels is limited by the paper type that is commercially available. We will solve these problems by further work and report them in due course.

## Methods

### Chemicals and materials

Nitrocellulose (NC) was purchased from China Sinopham (Shanghai, China). Double-sided adhesive tape with different colours and tools for fabricating the vPADs were all bought from local grocery store and supermarket. The 5-bromo-4-chloro-3‘-indolyphosphate p-toluidine salt/nitro-blue tetrazolium chloride (BCIP/NBT) substrate solution and substrate buffer solution, sodium dodecyl sulfate (SDS) were obtained from Sinopharm Chemical Reagent Co., Ltd (Shanghai, China). Coomassie brilliant blue, rhodamine B, methyl orange, bromocresol green, phosphate-buffered saline (PBS), polysorbate-20 (TWEEN-20), bovine serum albumin (BSA), Whatman grade 1 chromatography paper (20 cm × 20 cm) and colorimetric glucose assay kit was purchased from Sigma-Aldrich. The kits for colorimetric uric acid, cholesterol, triglyceride assays were purchased from Biosino Bio-Technology & Science Inc. (Beijing, China). Mouse anti-human myoglobin, human myoglobin, and alkaline phosphatase (ALP)-labelled goat anti-human myoglobin were purchased from Santa Cruz Biotechnology Co., Ltd (Shanghai, China). All solutions were prepared with deionised water (18.0 MΩ cm, Milli-Q Gradient System, Millipore) with ultraviolet sterilization. All reagents were used as received without further purification.

### Experimental Procedures

For multiplex colorimetric assays, two paper strips (Whatman grade 1 chromatography paper) were used to fabricate the vPAD, as shown in Fig. 2. Double-sided adhesive tapes with four different colours were attached to the paper for assembly of the device and also used as channel barrier. The color of the tape indicated the target being analysed in each channel (red: glucose, blue: uric acid, yellow: cholesterol, green: triglyceride). For the multiplex colorimetric assays, all of the reagents were from the assay kits and were prepared according to the instructions of the kit provider. To preload the assay reagents onto the vPAD, 5.0 µL aliquot of the reagent solution was dropcast into the detection reservoir at the end of each channel. The solution was allowed to dry at 20 °C under nitrogen. To initiate the multiplex colorimetric assay, 20 µL sample was introduced into the inlet at the centre of the device. After 20 min, an office scanner (HP C6180) was used to obtain optical images of the detection reservoirs, the images were imported into Adobe Photoshop CS5 to measure colour intensity. The detection of the four targets was based on a two-step reaction: (1) oxidase-catalyzed oxidation of targets/hydrolytic product of targets to yield H_2_O_2_ and (2) horseradish peroxidase-catalyzed oxidation of the substrate by H_2_O_2_ to yield a colored compound. The detection of glucose was based on glucose oxidase and o-dianisidine as the peroxidase substrate. The detection of uric acid was based on uricase and N-ethyl-N-(2-hydroxy-3-sulfopropyl)-3-methylaniline as the peroxidase substrate. For cholesterol, the detection was based on cholesterol esterase which catalyses the hydrolysis of cholesterol esters to yield free cholesterol. The peroxidase substrates used for cholesterol detection was 4-aminoantipyrine and phenol. The determination of triglycerides was based on hydrolysis of triglycerides by lipoprotein lipase to yield glycerol. The glycerol was then oxidised by oxygen which was catalyzed by glycerol oxidase. The peroxidase substrates involved in the final color-generating reaction were 4-aminophenazone and 4-chlorophenol.

For ELISA, 1.0 µL aliquot of 50 mM PBS (pH 7.4) containing 1 mg/mL mouse anti-human myoglobin antibody was prepared and then mixed with a 1.0 µL aliquot of 50 mM Na_2_HPO_4_ solution (pH 7.5) containing 1.0% (w/v) sucrose. 50 mM PBS (pH 7.5) solution containing 0.50% (w/v) BSA, and 50 mM phosphate buffer (pH 7.4) containing 0.010% (w/v) SDS were used for blocking and wash, respectively. 50 mM PBS (pH 7.5) containing 25 µg/mL ALP-labelled anti-human myoglobin antibody and 0.10% (w/v) BSA were prepared. The BCIP/NBT substrate solution was diluted 10 times with the substrate buffer solution. The 3D vPAD for ELISA was fabricated using two paper strips which were blocked with the blocking solutions (green and blue) and one NC strips (red), as shown in Fig. 3b. 1.0 µL aliquot of 0.50 mg/mL anti-human myoglobin solution was transferred to the NC strip (at location I in Fig. 3b) using a pipette having a cotton thread on its tip. The NC strip was allowed to dry for 1 hour, immersed in the blocking solution for 30 min, and finally washed by washing solution for 30 min at 25 °C. Double-sided adhesive tape (red) was attached to the NC strip for assembly of the device. 0.50 µL aliquot of 0.50 mg/mL ALP-labelled anti-human myoglobin antibody was dropcast onto one paper strip (at location II in Fig. 3b). 0.50 µL aliquot of BCIP/NBT substrate solution was dropcast onto the other paper strip (at location III in Fig. 3b). Both paper strips were allowed to dry completely at 25 °C and then attached to double-sided adhesive tape for assembly into the ELISA vPAD. To initiate the ELISA, aqueous sample containing myoglobin was introduced from the inlet. After 30 min, an office scanner (HP C6180) was used to obtain optical image of the detection reservoirs, the images were imported into Adobe Photoshop CS5 to measure colour intensity.
